# Intranasal Administration of Dolutegravir-Loaded Nanoemulsion-Based In Situ Gel for Enhanced Bioavailability and Direct Brain Targeting

**DOI:** 10.3390/gels9020130

**Published:** 2023-02-03

**Authors:** Anroop B. Nair, Sunita Chaudhary, Shery Jacob, Dhwani Patel, Pottathil Shinu, Hiral Shah, Ankit Chaudhary, Bandar Aldhubiab, Rashed M. Almuqbil, Ahmed S. Alnaim, Fatemah Alqattan, Jigar Shah

**Affiliations:** 1 Department of Pharmaceutical Sciences, College of Clinical Pharmacy, King Faisal University, Al-Ahsa 31982, Saudi Arabia; 2 Department of Pharmaceutics, Arihant School of Pharmacy & BRI, Adalaj, Gandhinagar 382421, India; 3 Department of Pharmaceutical Sciences, College of Pharmacy, Gulf Medical University, Ajman 4184, United Arab Emirates; 4 Department of Biomedical Sciences, College of Clinical Pharmacy, King Faisal University, Al-Ahsa 31982, Saudi Arabia; 5 Department of Quality Assurance, Saraswati Institute of Pharmaceutical Sciences, Gandhinagar 382355, India; 6 Department of Pharmaceutics, Institute of Pharmacy, Nirma University, Ahmedabad 382481, India

**Keywords:** NeuroAIDS, Dolutegravir, nanoemulsion, nasal in situ gel, mucoadhesive, brain targeting, optimization

## Abstract

Dolutegravir’s therapeutic effectiveness in the management of neuroAIDS is mainly limited by its failure to cross the blood–brain barrier. However, lipid-based nanovesicles such as nanoemulsions have demonstrated their potential for the brain targeting of various drugs by intranasal delivery. Thus, the purpose of this study was to develop a Dolutegravir-loaded nanoemulsion-based in situ gel and evaluate its prospective for brain targeting by intranasal delivery. Dolutegravir-loaded nanoemulsions were prepared using dill oil, Tween^®^ 80, and Transcutol^®^ P. Optimization of the nanoemulsion particle size and drug release was carried out using a simplex lattice design. Formulations (F1–F7 and B1–B6) were assessed for various pharmaceutical characteristics. Ex vivo permeation and ciliotoxicity studies of selected in situ gels (B1) were conducted using sheep nasal mucosa. Drug targeting to the brain was assessed in vivo in rats following the nasal delivery of B1. The composition of oil, surfactant, and cosurfactant significantly (*p* < 0.05) influenced the dependent variables (particle size and % of drug release in 8 h). Formulation B1 exhibits pharmaceutical characteristics that are ideal for intranasal delivery. The mucosal steady-state flux noticed with BI was significantly greater (*p* < 0.005) than for the control gel. A histopathology of nasal mucosa treated with BI showed no signs of toxicity or cellular damage. Intranasal administration of B1 resulted in greater C_max_ (~six-fold, *p* < 0.0001) and AUC_0−α_ (~five-fold, *p* < 0.0001), and decreased T_max_ (1 h) values in the brain, compared to intravenous administration. Meantime, the drug level in the plasma was relatively low, suggesting less systemic exposure to Dolutegravir through intranasal delivery. In summary, the promising data observed here signifies the prospective of B1 to enhance the brain targeting of Dolutegravir by intranasal delivery and it could be used as a feasible and practicable strategy for the management of neuroAIDS.

## 1. Introduction

Acquired immunodeficiency syndrome (AIDS) is a global health threat, which is caused by a specific etiologic agent human immunodeficiency virus (HIV) [1]. The causative retrovirus targets the immune system directly or indirectly, which reduces patients’ resistance to various diseases. Thus, the infected individuals gradually become immunodeficient. One of the main locations where HIV-1 resides is in the central nervous system (CNS), where it can survive for an extended period of time. NeuroAIDS are the neurological complication associated with AIDS and certain neuropsychiatric issues are also registered in AIDS patients [2]. These clinical manifestations continue to be a serious problem for patients with chronic HIV infection, particularly children and patients with poor adherence. Nevertheless, drug resistance, toxicity, and viral reservoirs make the lifelong treatment of HIV infection challenging. Although current anti-HIV medications are effective at lowering plasma viral loads, they are unable to completely eradicate the virus from the brain. This is mainly because of the low antiretroviral drug perfusion into the brain, resulting in insufficient drug levels at the target, primarily due to the tight junctions in the blood–brain barrier [3]. Moreover, bioavailability is significantly affected by P-glycoprotein/P-gp multidrug efflux transporters that are particularly expressed at the blood–brain barrier, for example, CYP 450-mediated biotransformation, besides rapid elimination [4]. Hence, alternative dosage forms are necessary to provide brain targeting, which improves bioavailability, higher permeability, and the reduced drug efflux.

Integrase strand transfer inhibitors (INSTIs) are oral retroviral agents that are crucial in HIV treatment. The therapeutic efficiency and safety of INSTIs in HIV-infected patients were established in multiple studies [5,6,7]. An essential part of HIV replication called HIV integrase is inhibited by INSTIs. Based on the current practice guidelines, INSTIs are one of the first-line therapies for HIV-infected patients, regardless of the pre-treatment viral load. Dolutegravir is indeed a second-generation INSTI that has excellent advantages, including a low risk of drug–drug interactions, a once-daily dose, and a high genetic barrier to drug resistance [8]. This drug is well-tolerated and possesses a good safety profile, a powerful antiviral effectiveness, and a quick virus inhibition [9,10,11,12].

Nanoemulsions are thermodynamically unstable, transparent, or translucent colloidal dispersion systems that are typically composed of an isotropic mixture of oil, surfactant: cosurfactant (S_mix_), water, and drugs [13]. The dispersed phase droplet size ranges between 50 and 500 nm, which has a very low oil and water (o/w) interfacial tension [14]. Extensive studies were conducted to explore the feasibility of nanoemulsions in several drug delivery systems such as passive targeting and for the treatment of various cancers [15]. The literature indicates that nanoemulsions encapsulated with a protease inhibitor drug, namely indinavir, have been successfully used for brain targeting, probably due to lipoprotein-mediated endocytosis and P-gp inhibition [16].

Intranasal administration is a non-invasive technique that delivers medications to the brain (bypassing the blood–brain barrier) along the olfactory and trigeminal neural pathways, limiting systemic exposure and side effects. The drug is delivered via the nasal route directly to the brain by preparing a nanoemulsion of Dolutegravir, utilizing an olfactory pathway, which is expected to be more efficient and will give promising therapeutic effects in the management of NeuroAIDS. In situ polymeric gel formulations are in patient-friendly droppable sol forms that can transform into a viscoelastic gel when exposed to normal physiological conditions. The general theory behind the sol–gel transition is based on the influence of different external stimuli, such as pH, temperature, solvents, ultraviolet irradiation, and the presence of distinct ions or molecules [17]. Due to the practical feasibility, temperature-responsive hydrogels are the most frequently used polymer systems that undergo in situ-gel formation when they are exposed to the normal physiological temperature of the nasal mucosal membrane [18].

Poloxamer 407 is a synthetic, non-ionic triblock copolymer that undergoes a phase transition when the temperature is changed. This polymer possesses beneficial properties, such as good aqueous solubility, minimum toxicity/tissue sensitivity, solubilization capability, mucoadhesion, and control release of the drug, and is also compatible with various types of actives and excipients [19,20]. On the other hand, various lipid-based, inorganic, and polymeric nanoparticles can provide personalized medicine in drug delivery [21,22]. However, the real potential of liposome formulations requires multifunctional designs [23,24]. The benefits of using poloxamer 407-based nanocarriers include their ability to form stable, thermoreversible hydrogels, and possess good biocompatibility. The high water content of a poloxamer gel can cause hydration of the nail plate, which in turn can aid in the transungual delivery and extend the duration of nanoparticle retention inside the nail plate [25]. Compared to clinically used liposomes and human serum albumin, poloxamer-based nano-carriers have an improved stability, a lower toxicity, and better targeting capabilities, and they were investigated in various drug delivery applications including cancer therapy, gene delivery, and wound healing [26].

Poloxamer’s thermoreversible characteristic is mostly a result of its poor solubility in block copolymer micelles. The ethylene oxide and propylene oxide chains in the poloxamer 407-copolymer interact hydrophobically to cause thermogelation. By increasing the temperature, the hydrophobic propylene oxide repeat units begin to dehydrate and the poloxamer 407 copolymer chains begin to combine into a micellar structure, which eventually leads to gelation [27]. Poloxamer may not be able to offer sufficient mechanical strength and mucoadhesion on its alone; hence, other mucoadhesive agents are generally incorporated into the gel formulation. This would increase the retention of formulations at the nostrils and thereby prolong the duration of the drug release and subsequent therapeutic effect [28]. The most popular mucoadhesive ingredient for making in situ gel formulations is carbopol (polymer), because of its exceptional mucoadhesive characteristics [29]. This study aimed to formulate a Dolutegravir-loaded nanoemulsion and assess its achievability for nose-to-brain delivery. The optimization of nanoemulsions was performed by a simplex lattice design. A selected nanoemulsion was incorporated in a thermosensitive gel (B1) and assessed for its brain-targeting ability in rats.

## 2. Result and Discussion

### 2.1. Saturation Solubility Study

**Figure 1:**
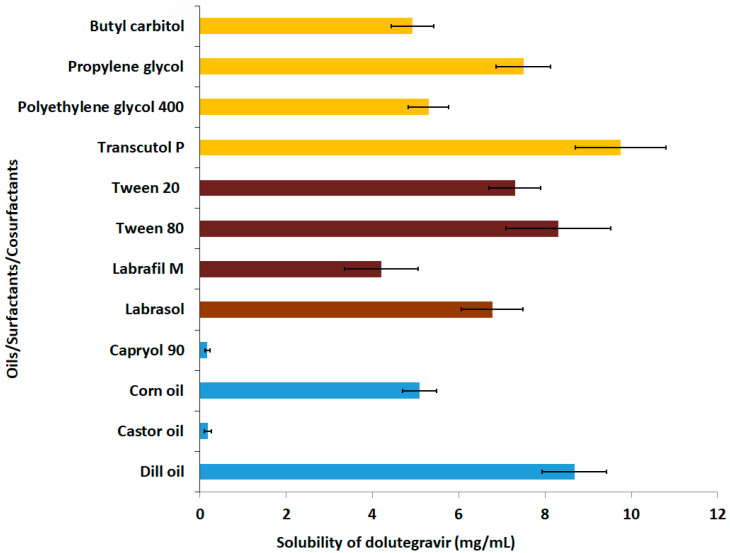
Dolutegravir solubility determined in various oils, surfactants, and cosurfactants. The value mentioned is average ± SD (n = 6).

The selection of suitable vehicles is crucial for the development of a stable nanoemulsion [30]. Preliminary experiments were performed to assess the solubility of Dolutegravir to identify suitable solvents for the drug. The solubility of Dolutegravir was estimated in different oils, surfactants, and cosurfactants, and is depicted in Figure 1. Based on the greater solubility data (Figure 1), the oil phase (dill oil), surfactant (Tween^®^ 80), and cosurfactant (Transcutol) were chosen for the nanoemulsion formulation. The beneficial effect of the above-mentioned vehicles in formulating nanoemulsions is reported earlier. For instance, dill oil has been used in nanoemulsions due to its ability to enhance the solubility of hydrophobic drugs [31]. Similarly, Tween^®^ 80 has been used as a surfactant in many nanoemulsion preparations, which improved their encapsulation efficiency and stability [32]. Tween^®^ 80 is extensively used as an emulsifying agent in nanoemulsions [33] and as a co-emulsifying agent for the reduction of the droplet size in lipid emulsions [34]. Moreover, Tween^®^ 80 has been used in nanoparticles to enhance the targeted brain delivery of drugs [16]. Similarly, Transcutol has demonstrated improved nasal penetration of certain drugs in brain-targeted drug delivery [35].

### 2.2. Pseudo Ternary Phase Diagram

**Figure 2:**
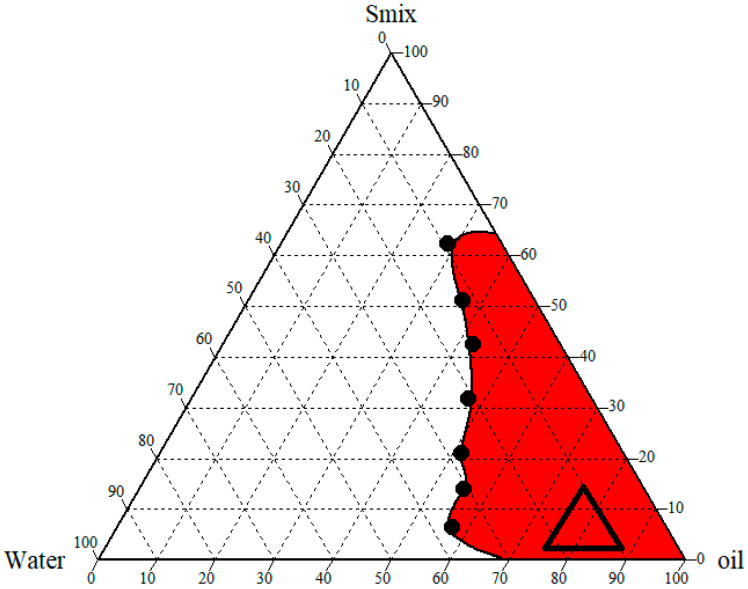
Ternary phase diagram showing nanoemulsion region prepared using dill oil, Tween^®^ 80: Transcutol (S_mix_), and water.

To develop nanoemulsions with the best stability, the most suitable concentration of oil, water, and ideal Smix ratio was determined using a ternary phase diagram. Based on the high transparency (99.3%, Table S1) of the dispersion, the S_mix_ ratio between the surfactant and cosurfactant was fixed at 1:1. Figure 2 shows the pseudo-ternary phase diagram of the systems comprising of a surfactant: cosurfactant mixture (*S*_mix_), oil, and water. The physical appearance of the sample shifting from either turbid to transparent or vice versa was used to mark the binodal curve between the two phases in the ternary phase illustration. By using the water titration approach, the ratios of the oil, S_mix_, and other components were changed to complete the whole nanoemulsion domain. It was noted that formulations with the surfactant and the cosurfactant ratio of 1:1 produced stable emulsions (at 48 h). The ternary phase diagram’s findings also demonstrated that the formulation’s turbidity grew along with the oil concentration. This might be explained by an increase in interfacial tension between both the aqueous and oil phases because of an insufficient surfactant system concentration.

### 2.3. Optimization of Nanoemulsion

The mixture of the oil, water, and S_mix_ that made up the nanoemulsion was optimized using a simplex lattice design. These three components were tested by varying their concentrations simultaneously while maintaining the total volume constant. The drug was dispersed in the dill oil by ultrasonication. The nanoemulsion formulations were formulated by the aqueous titration method. Table 1 shows the composition of design batches and responses of the dependent variables.

**Figure 3:**
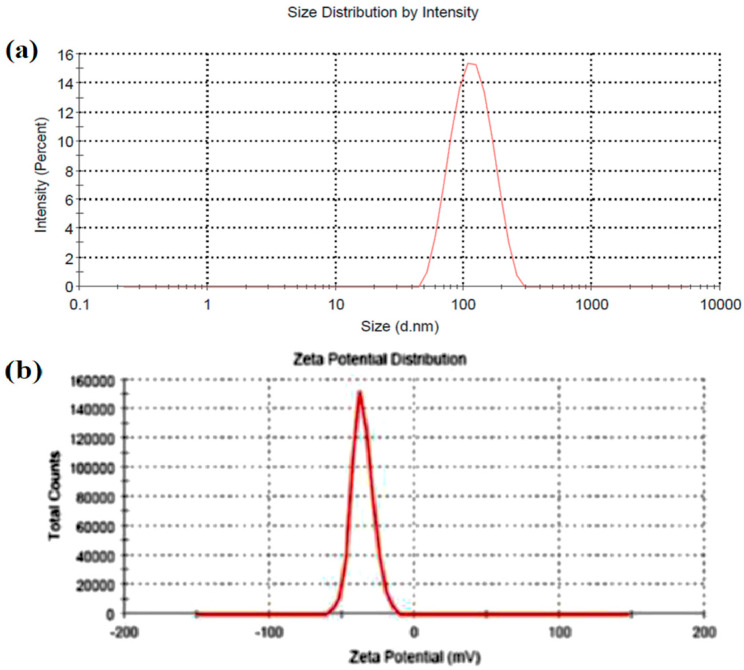
Observed particle size distribution (**a**) and zeta potential (**b**) of optimized nanoemulsion (F3) by dynamic light scattering technique.

The particle size of nanoemulsions is generally utilized to assess the stability of formulations as the instability of the emulsion is related to particle coalescence, coagulation, and flocculation. Moreover, particle size is one of the crucial properties assessed in the evaluation of nanoemulsions to ensure their suitability for intranasal delivery. The particle size of the prepared nanoemulsions (F1–F7) ranged from 106.36 to 286.65 nm, demonstrating the nanosized range (Table 1). The results of the particle size analysis of nanoemulsion F3 are depicted in Figure 3, which shows that the distribution of the particle sizes was roughly Gaussian, unimodal, and narrow.

The percentage release value ranges between 61.08 and 95.80%, as shown in Figure S1. Formulation F3 showed the smallest particle size with the highest % drug release, and formulation F6 showed the highest particle size with the smallest % drug release. The particle size and % drug release values for the formulations (F1 to F7) showed an extensive variation. The statistics show that the independent factors have a significant impact on the particle size and % drug release values.

#### 2.3.1. Statistical Analysis

Effect of X_1_, X_2_, and X_3_ on Particle size

The following equation represents the fitted full model equation connecting the response (Y_1,_ particle size) to the transforming factor.

Y_1_ = 286.24X_1_ + 106.02X_2_ + 184.44X_3_ + 186.54X_1_X_2_ +70.45X_1_X_3_ − 99.81X_2_X_3_.

A higher value of r^2^ = 0.99 showed a good agreement between the dependent and independent variables. The significance level of the coefficient ß_1_, ß_2_, ß_3_, and ß_12_ terms were lesser than 0.05 (*p*-value); therefore, these values were kept in the full model as well to create a reduced model. Thus, X_1_, X_2_, X_3_, and X_12_ have a substantial impact on Y_1_; hence, the reduced model equation is as follows:

Y_1_ = 286.24X_1_ + 106.02X_2_ + 184.44X_3_ + 186.54X_1_X_2_

**Figure 4:**
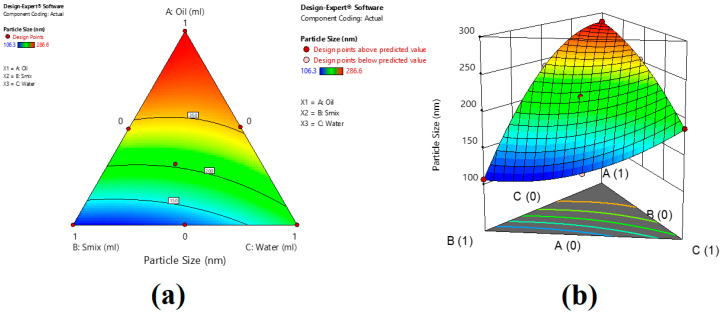
Counter (**a**) and 3D surface plot (**b**) of the effect of the independent variables on particle size.

The design expert program performed the statistical analysis of the findings for the particle size of all the formulations prepared. After examining the F statistics findings, it was found that the model’s probability was more than the F value (361), which supports the model’s importance. The model’s significance was established, as the *p*-value was less than 0.05. The model terms X_1_, X_2_, X_3_, and X_1_X_2_ are found crucial in this situation. As the coefficient of X_1_, X_2_, and X_3_ are positive, dill oil, Smix, and water increase the particle size with their proportion. X_1_X_2_ also has a synergistic effect on the particle size, which is in agreement with the literature [36]. Figure 4 shows Counter and 3D surface plots of the effect of the independent variable on particle size.

Effect of X_1_, X_2_, and X_3_ on the % Drug release in 8 h

The following equation represents the fitted full model equation connecting the response (Y_2_, % drug release) to the transforming factor.

Y_2_ = 61.06X_1_ + 95.79X_2_ + 80.21X_3_ − 8.94X_1_X_2_ − 1.72X_1_X_3_ − 7.74X_2_X_3_.

A higher value of regression (r^2^ = 0.99) showed a good agreement between the dependent and independent variables. The significance level of the coefficient ß_1_, ß_2_, ß_3_, ß_12_, and ß_23_ terms were <0.05 (*p*-value); therefore, these values were kept in the full model as well to create a reduced model. Thus, X_1_, X_2_, X_3_, X_12_, and X_23_ have a significant effect on the dependent variable (Y_2_); hence, the reduced model equation is as follows:

Y_2_ = 61.06X_1_ + 95.79X_2_ + 80.21X_3_ − 8.94X_1_X_2_ − 7.74X_2_X_3_.

The design expert program performed the statistical analysis of the findings for the particle size of all the formulations prepared. After examining the F statistics findings, it was found that the model’s probability was more than the F value (3477), which supports the model’s importance. The model’s significance was established, as the *p*-value was less than 0.05. The model terms X_1_, X_2_, X_3_, X_12_, and X_23_ are found to be crucial in this situation.

**Figure 5:**
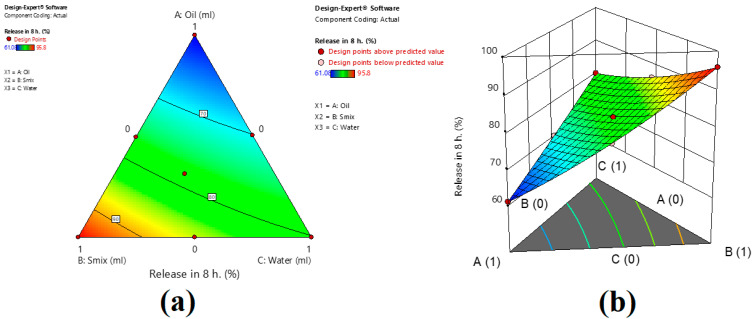
Counter (**a**) and 3D surface plot (**b**) of the effect of the independent variable on % drug release in 8 h.

From the contour plot, it can be revealed that the amount of S_mix_ (Tween^®^ 80: Transcutol) has a higher effect on the % drug release compared to dill oil and water. X_1_X_2_ and X_2_X_3_ have a negative value of coefficient, which indicated the antagonistic effect on drug release from the nanoemulsion and is in agreement with an earlier report [37]. The effect of the independent variable on the % drug release is depicted in Figure 5 as a Counter and 3D surface plot.

#### 2.3.2. Validation of the Model

**Figure 6:**
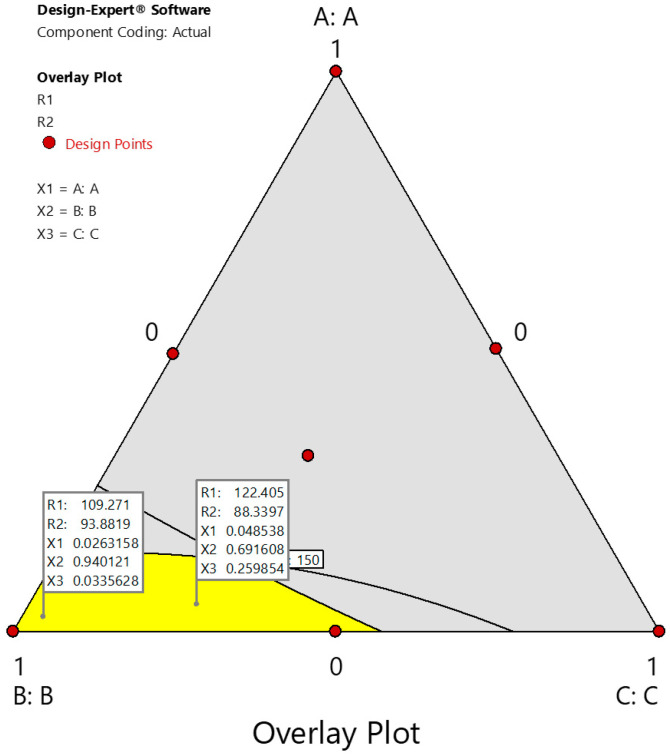
Overlay plot for the simplex lattice design batches.

An overlay plot of all three variables, dill oil (X_1_), Smix (X_2_), and water (X_3_), was drawn to analyze the particle size and the % drug release. The plot was drawn using design expert software (v.11.0). From the overlay plot (Figure 6), the design space and two points (formulations F8 and F9) were selected as checkpoint batches to validate the design model. The dependent parameters, i.e., particle size and the % drug release were determined and compared with predicted values, as shown in Table 2. The results obtained with checkpoint formulations are very close to the predicted values. Thus, it can be concluded that the statistical model is mathematically valid.

### 2.4. Characterization of Nanoemulsions

#### 2.4.1. Percentage Transmittance and Phase Separation

All the prepared formulations (F1–F7) were transparent and there was no phase separation, suggesting that the nanoemulsions are stable.

#### 2.4.2. Polydispersity Index and Zeta Potential

A polydispersity index value < 0.5 denotes spherical vesicles that are homogeneous, consistently sized, and sphere-shaped. The polydispersity values for F1–F7 varied from 0.181 to 0.540, indicating that the samples were relatively homogeneous and regular. Formulations F1, F3, F6, and F7 demonstrated polydispersity index values of 0.249, 0.202, 0.326, and 0.181, respectively, which shows uniformity in the particle size distribution. However, F2, F4, and F5 formulations with polydispersity index values of 0.505, 0.540, and 0.441, respectively, displayed near heterogeneously dispersed globules.

Zeta potential is another parameter that indicates the electrostatic charge between emulsion droplets and the continuous phase. A large zeta potential value (typically > ± 30 mV) shows enough repellency between particles with like charges to prevent flocculation or agglomeration and possibly even stabilize the dispersion, following the conventional electrical double-layer theory. Higher zeta values noticed (−36.5 to −42.9 mV) with prepared formulations (F1–F7) can provide a strong repulsive force between the nanoemulsion particles, which could eventually result in good stability and prevent accumulation. A representative image of the zeta potential measured with formulation F3 is presented in Figure 3. Moreover, the measured surface charges of nanoemulsions did not drastically change, indicating that the formulation’s constituents did not affect the zeta potential values.

#### 2.4.3. Transmission Electron Microscopy (TEM)

**Figure 7:**
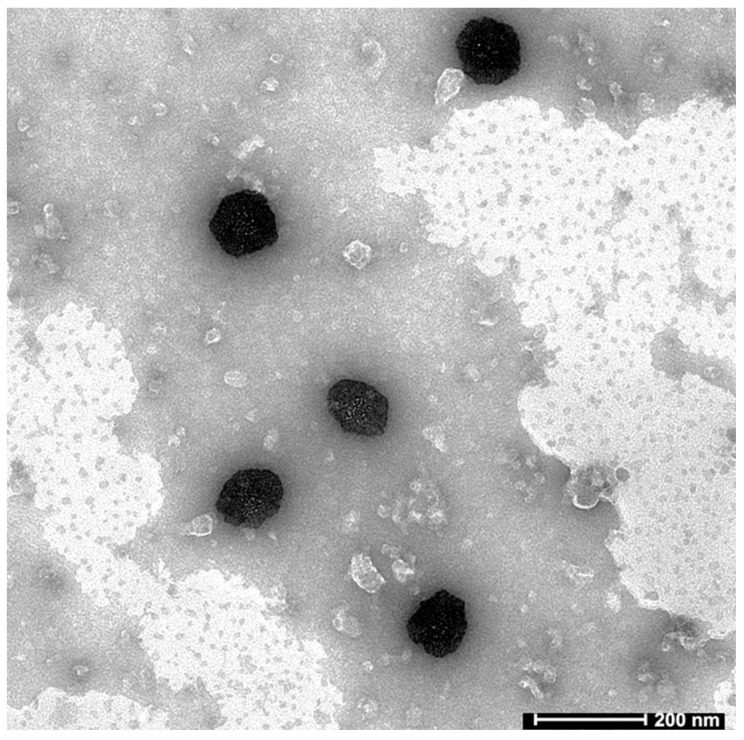
A representative TEM image of selected Dolutegravir-loaded nanoemulsion (F3).

Figure 7 displays a typical TEM micrograph of the chosen nanoemulsion (F3). According to a visual analysis of the image, the nanoemulsion particles were spherical, uniformly distributed, immediately distinguishable, and had uniform surfaces that were free of any aggregation or coalescence. The particles were randomly spread in the continuous phase without agglomerating and looked darker against a bright background. The cluttered background seen in the image could be due to specimen thickness that caused a fogging effect, or the sample itself could have had a high background noise [38]. The particle size was determined to be between 100 and 200 nm. The range of particle sizes seen in Figure 7 and the results of the particle size analysis were in excellent agreement.

### 2.5. Evaluation of Formulated Gels

#### 2.5.1. Appearance, pH, and Drug Content

Gels (B1–B6) formulated with the optimized Dolutegravir-loaded nanoemulsion (F3) were analyzed for physical parameters such as transparency, pH, and drug content, and the results are tabulated in Table 3. The clarity of all the gel formulations without the presence of any particulate matter was confirmed by visual observation. It was reported that human nasal mucosal pH approximately ranges between 5.5 and 6.5; therefore, nasal formulations with said ranges can potentially avoid any nasal sensitization [39]. The pH of all the gels was in the range of 5.2 to 5.9, and there was no significant variation between the formulations. The pH results also showed that there was a trend that the increase in poloxamer quantity slightly increased the pH, while the inclusion of more carbopol content moderately reduced the formulation pH, as reported earlier [40]. A higher drug content was noticed with all the formulations tested, demonstrating a reasonable retention of Dolutegravir within the polymer matrix and further proving that the composition of the gel did not significantly affect the content uniformity (Table 3).

#### 2.5.2. Gelation Temperature

In the case of the thermoreversible gel, liquid sol is changed into a gel phase triggered by an external stimulus and the temperature. The ideal gelation temperature (between 25 °C and 32 °C) of a product could overcome the typical formulation and drug delivery challenges associated with in situ gels during production, storage, handling, and the time of application [41]. The gelation temperature noticed with B1-B6 was between 25 and 30 °C (Table 3). An evaluation of the data provided in Table 3 demonstrates that the gelation temperature of B1-B6 considerably (*p* < 0.05) decreased when the concentration of poloxamer was raised from 20 to 22%. It was reported in earlier investigations that the presence of certain other chemicals in the gels could affect the poloxamer gelation temperature [42,43]. The observed data here signified that the gelation temperature of the gels was also influenced (*p* < 0.05) by the rise in the carbopol amount (0.1 to 0.3% *w*/*v*). According to the results, the formulations containing 20% poloxamer (B1–B3) demonstrated the gelation temperatures (28–30 °C) required for nasal administration.

#### 2.5.3. Viscosity

The rheological characteristics such as viscosity may significantly influence the nasal residence time and diffusion rate of the drug. The viscosity of a formulation mainly depends on its physicochemical characteristics, and on the temperature conditions to which it is applied. Carbopol 940 and poloxamer 407 polymers used as gelling agents play a predominant role in controlling the viscosity of the gel preparations [44]. The viscosity of developed gels (B1–B6) ranged from 2900 to 7300 cPs (Table 3). The data observed in Table 3 show that the rise in viscosity was influenced by the mucoadhesive carbopol polymer and it increases with an increase in the polymer content when compared with the thermosensitive polymer used (poloxamer). This observation is also in agreement with an earlier study [45].

### 2.6. In Vitro Drug Release

**Figure 8:**
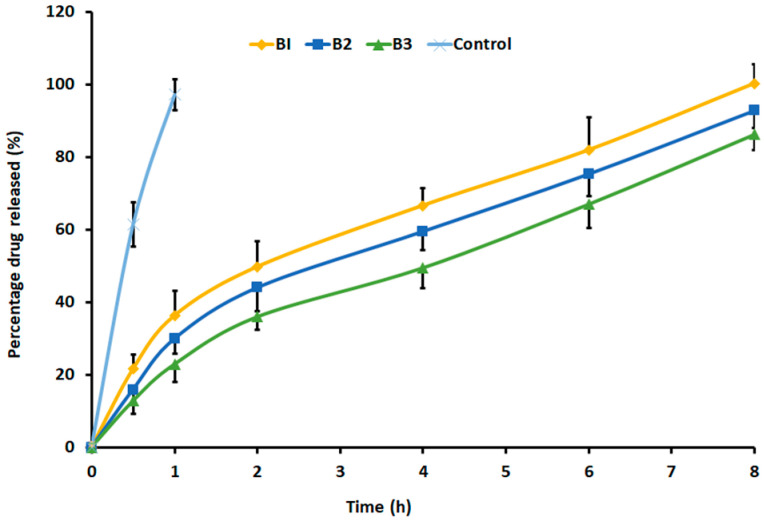
Dolutegravir release profiles from gel formulations (B1, B2, and B3) and control gel. The value mentioned is average ± SD (n = 6).

The release of drugs from the gels is a crucial factor that relates to the effectiveness of the product and could be correlated to the in vivo performance [46]. Figure 8 displays entirely different release profiles for the in situ gel (B1–B3) as compared to the control. A greater, controlled, and extended-release of drugs was demonstrated in B1, endorsing the gel’s ability to deliver drugs steadily for up to 8 h. It was evident from the figure that an increase in the quantity of carbopol in the formulated gel causes a reduction in the cumulative amount of Dolutegravir release. These results could be correlated to the cross-linked structure of the carbopols that form a hydrogel, which generally exist as microgels. Consequently, increasing the amount of carbopol tends to provide a slower and linear drug release that gradually diffuses through a moistened polymer matrix. This is probably because the gel structure acted as a resilient boundary to drug release. It is documented that the decrease in the number and size of water canals as well as the rise in the density of the 3D network inside the gel structure may account for this higher resistance [47]. In the control (gel), the release was quick and the whole drug reached the receiver within 1 h, probably due to the absence of any diffusion barrier. Formulation B1 was chosen for additional ex vivo and in vivo investigations because the drug release was higher and complete.

### 2.7. Ex Vivo Permeation Studies

**Figure 9:**
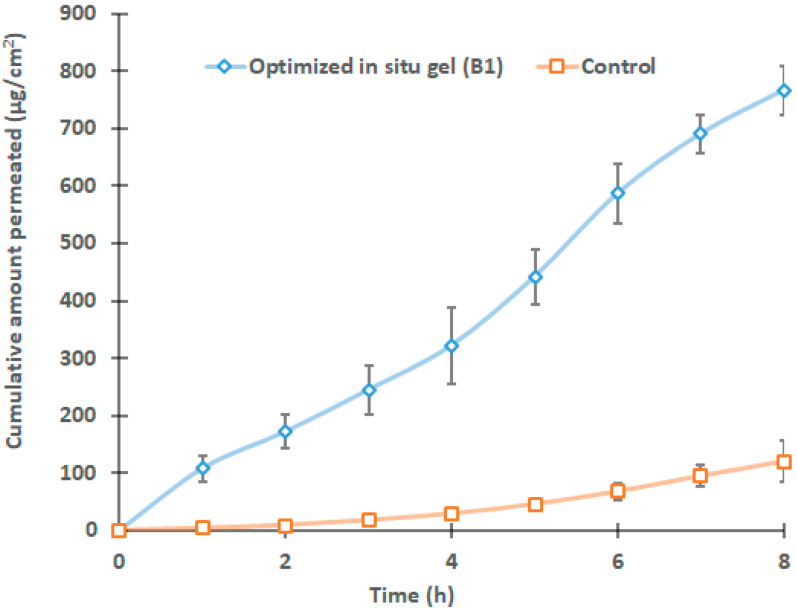
Comparison of drug permeation of selected in situ gel (B1) and control gel across sheep nasal mucosa. The value mentioned is average ± SD (n = 6).

Membrane permeation experiments are typically carried out using a formulation against similar animal membranes to understand the potential behavior in real-time [48]. The physicochemical properties of the actives besides the anatomy and physiology of the membrane bordering the targeted site mainly control the drug transport. A sheep nasal mucosa membrane that is quite comparable to the human [49] was used to assess the drug penetration from the selected gel (B1) and control. The profiles in Figure 9 indicate greater Dolutegravir permeation (*p* < 0.005) from formulation BI in comparison to the control. Indeed, a six-fold increase in flux (~97.32 μg/cm^2^/h) was noticed with BI when compared with the control gel. The lag time noticed with the in situ gel and control were ~0.196 h and 1.12 h, respectively. The higher drug permeation (~766.37 μg/cm^2^) demonstrated by BI indicates the potential of nanoemulsion carriers to transport easily into and through the nasal mucosal layers. In contrast, the low flux values (~15.01 μg/cm^2^/h) of the control gel could be due to the low intrinsic permeability of the Dolutegravir. Overall, the findings here suggest that the selected formulation B1 administered via the nasal route may increase the direct brain delivery of Dolutegravir.

### 2.8. Nasal Ciliotoxicity

**Figure 10:**
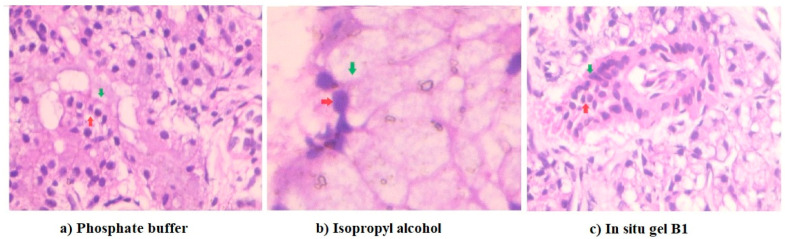
Histopathological analysis of nasal mucosa of sheep. Nasal mucosa exposed to phosphate buffer (**a**) and in situ gel B1 (**c**) were comparable and did not show any signs of toxicity or cellular damage, demonstrating the ability of the formulation to maintain cellular integrity. The cellular morphology of the nasal mucosa was severely harmed by the isopropyl alcohol treatment (**b**).

Histopathological images of various treatments in nasal ciliotoxicity studies using sheep nasal mucosal membranes are illustrated in Figure 10. The microscopic pictures of nasal mucosa exposed to a phosphate buffer (Figure 10a) show unaltered nasal cells with intact basement membranes (red arrows), as well as unharmed lobular cells with distinct nuclei (green arrows). Similarly, the treatment of the selected in situ gel of Dolutegravir (B1) with nasal tissues also showed an undamaged basal layer (red arrow) and a normal lobular cell organization with well-defined nuclei (green arrow) (Figure 10c). However, treatment with isopropyl alcohol caused substantial cellular and histological damage to the nasal mucosa, resulting in the disruption of the basal cells (red arrow) and the destruction of the lobular cells’ nuclei, resulting in the formation of multinucleated cells (green arrow) (Figure 10b). Overall, the data here suggest the safety of the selected in situ gel for nasal application.

### 2.9. Animal Studies

**Figure 11:**
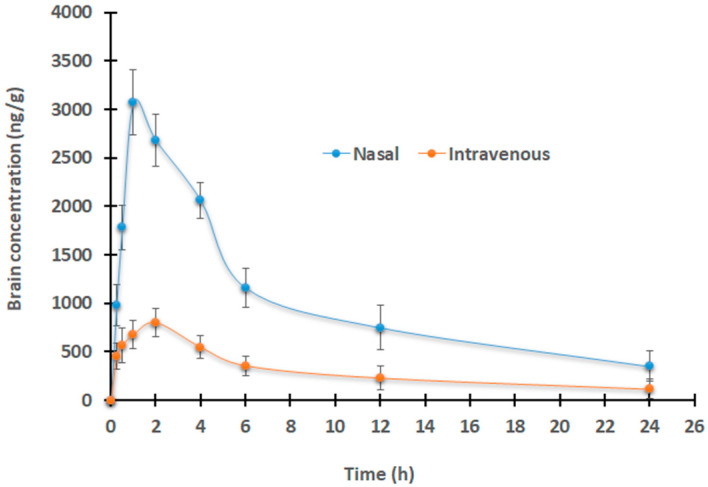
Comparison of drug brain levels following single-dose of Dolutegravir (150 µg/rat) after intravenous injection (0.4 mL) or selected gel (B1, 30 µL) by nasal route. The value mentioned is average ± SD (n = 6).

The main aim of this investigation was to assess the enhancement of the brain-targeting ability and the overall bioavailability contributed by developing a Dolutegravir-loaded nanoemulsion in a patient-friendly, droppable in situ nasal gel formulation. In general, the drug concentration in the brain is restricted by the highly selective barrier and is also dependent on various physicochemical factors of the drug. Therefore, the brain drug concentration–time profiles may be markedly different from the plasma drug concentration contributed by various influx and efflux clearance mechanisms. Since the drug undergoes various distributional and elimination processes within the brain, quantitative evaluation through pharmacokinetic modeling is essential to anticipate its effect. A refined mathematical model is essential to predict the spatial drug distribution within the brain besides locoregional differences that allow for the integration of various physiological parameters. Figure 11 shows the drug brain concentration profiles following a single-dose of Dolutegravir (150 µg/rat) given by an intravenous injection (0.4 mL), or a selected gel (B1, 30 µL) by nasal route. The various pharmacokinetic properties measured are summarized in Table 4. A significantly different (*p* < 0.005) brain drug concentration–time profile was observed after intranasal administration of nanoemulsion-based in situ gel when compared to an equivalent dose administered through the intravenous route. As expected, absorption via the nasal route was very quick, with a higher Dolutegravir entering the brain (~726.34 ng/g, 15 min) in comparison to its counterpart (~221.39 ng/g). It was reported previously that the nasal therapy of talinolol-loaded nanoemulsions demonstrated a greater brain level in rats [50]. Intranasal administration of B1 resulted in a lesser T_max_ value (1 h), while it was relatively higher (2 h) with intravenous administration (Table 4), indicating the rapid delivery of Dolutegravir by the nasal route. Furthermore, nose-to-brain delivery of nanoemulsion gel demonstrated a ~6-fold high C_max_ value (2274.75 ng/g) when compared to its counterpart (387.42 ng/g).

The increased Dolutegravir penetration from the designed in situ gel-based nanoemulsion could be another explanation for the greater C_max_ values observed in the intranasal delivery. The greater values in all of the pharmacokinetic properties observed during the intranasal delivery of Dolutegravir show that the selected in situ gel (B1) can have extremely good contact with the mucosal surface and is therefore held in the nostril for a longer period for sustained therapy. The brain drug amount appears to be declining gradually in both regimens, probably due to the long half-life of Dolutegravir. The higher bioavailability of Dolutegravir in the brain is demonstrated by a higher AUC_0−α_ (Table 4), which confirms the higher intake of Dolutegravir from the nanoemulsion-based gel. It was postulated that nanoemulsions have the greater flexibility to diffuse through the endothelial lining, resulting in enhanced retention in brain capillaries and producing a greater drug concentration gradient through endothelial cells [16]. It was also suggested that endocytosis and transcytosis could be the probable mechanism responsible for the passage of nanoemulsions into the brain due to their lipophilic characteristics. A comparatively smaller quantity of Dolutegravir that enters the brain via intravenous administration would probably be because of the limited permeation of the Dolutegravir from circulating plasma to highly selective semi-permeable endothelial cells bordering the tight junctions of the brain, as described elsewhere [51]. The literature revealed that mucoadhesive intranasal nanoemulsion can enhance the drug uptake into the brain, AUC, and hence proved its brain-specific targeting capability [52]. The relative bioavailability of Dolutegravir, measured by AUC, was found to be five times higher in the brain than in plasma (Table 4), supporting the theoretical and practical viability of the nose-to-brain approach using nanoemulsion-based in situ gel for CNS delivery. Indeed, this study indicates that the brain’s bioavailability of Dolutegravir was significantly increased when the formulated in situ gel was administered through the nasal route.

**Figure 12:**
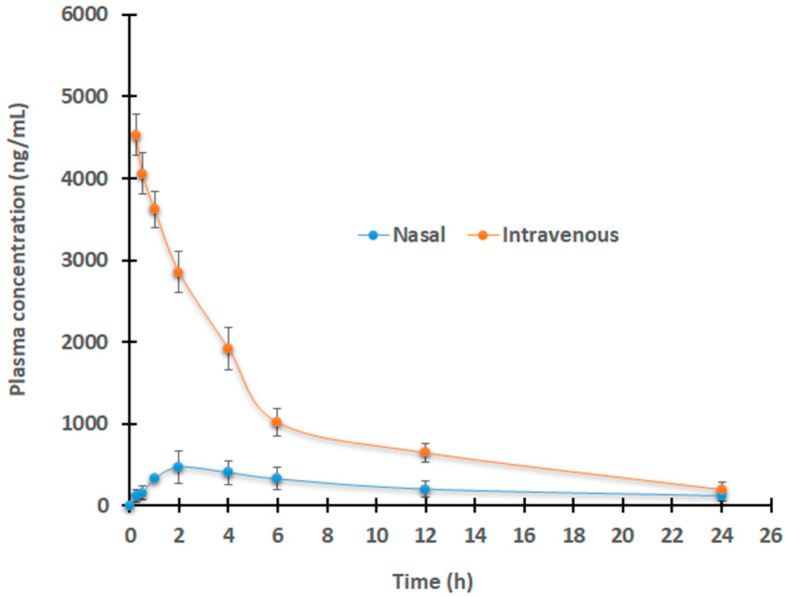
Comparison of plasma drug levels following single-dose of Dolutegravir (150 µg/rat) after intravenous injection (0.4 mL) or selected gel (B1, 30 µL) by nasal route. The value mentioned is average ± SD (n = 6).

In the meantime, the drug level in plasma was also measured to check the Dolutegravir availability in the central compartment after the administration of the solution by the intravenous route and in situ gel by the nasal route. Figure 12 shows that an intravenous mode of delivery resulted in a higher drug concentration in the plasma than nasal therapy. The computed AUC_0−α_ for the intravenous injection was 5.4 times greater (*p* < 0.0001) as compared to nasal delivery. In addition, intranasal drug delivery resulted in a low drug plasma concentration–time profile, which suggested that Dolutegravir was only little exposed systemically via the paracellular transport pathway. The low drug absorption into the central compartment could be advantageous as this could reduce the concentration of drugs at a site other than the target organ, hence, the low toxicity.

### 2.10. Stability Study

An accelerated stability was performed for the selected B1 in situ gel formulation. Indeed, there were no substantial changes in all the parameters tested during the stability period of three months. The drug release profile of the B1 formulation before and after the stability study was found to be similar when compared using a t-test that considered two samples with equivalent variances. After three months, the chosen B1 in situ gel formulation shows no statistically significant difference because the observed *t*-test value of 0.263 is far lower than the t-critical value of 1.78. In summary, the data indicates that the gel formulation did not exhibit any appreciable changes in any of the characteristics examined over three months while stored at 25 °C.

## 3. Conclusions

The nose-to-brain delivery is considered a promising drug-targeting approach for the effective transport of therapeutics to the brain for CNS disorders. In this context, developing a patient-compatible intranasal formulation such as nanoemulsion-loaded in situ gel could be ideal for the treatment of NeuroAIDS. This in situ gel formulation can improve drug delivery to the CNS by enhancing the formulation retention in the nasal cavity and thereby enhancing the drug transport. Hence, in the current study, a Dolutegravir-loaded nanoemulsion in situ gel was formulated (BI) and assessed for its nose-to-brain delivery. The Dolutegravir-loaded nanoemulsion was optimized by varying the concentrations of dill oil, Tween^®^ 80, and Transcutol^®^ P. A series of nanoemulsions (F1-F7) were used for the assessment of the particle size and drug release. The optimized nanoemulsions (F3) consisting of oil (5% *v*/*v*), S_mix_ (25% *v*/*v*), and water (70% *v*/*v*) possess small particles and exhibited a faster release profile. The optimized formulation (F3) was modified into in situ gels (B1–B6) using thermoresponsive (poloxamer 407) and mucoadhesive polymers (carbopol 934P). The selected Dolutegravir-loaded nanoemulsion in situ gel (B1) demonstrated acceptable pharmaceutical characteristics and was found to be safe for nasal application. The higher drug level in the brain after nasal administration confirms that the in vivo investigation supports the ex vivo data. The data here demonstrated the efficacy of selected in situ mucoadhesive gel formulations to directly deliver Dolutegravir into the brain through the intranasal route, which could provide an effective strategy to eradicate HIV from the brain, hence the treatment of NeuroAIDS. In conclusion, intranasal delivery using nanoemulsion incorporated into thermosensitive gels offers a promising approach for CNS targeting, and could be utilized for the effective treatment of various neurologic disorders.

## 4. Materials and Methods

### 4.1. Materials

Dolutegravir (MW, 419.38 Da, and 99.99%) was obtained as a gratis sample from Emcure Pharmaceuticals, Gandhinagar, India. Transcutol^®^ P, Capryol^®^ 90, Labrasol^®^, and Labrafil^®^ M were purchased from Gattefosse, Saint-Priest, France, and were of USP-NF/EP quality. Dill oil (95.9%) and corn oil (92.3%) were gifted from Vicci Win Pharma, Ahmedabad, India. Castor oil (88.5%) was gifted from Gujarat Glycols, Ankhleswar, India. Polyethylene glycol (PEG) 400 (98%), butyl carbitol (92%), and propylene glycol (99%) were gifted from Himedia, Mumbai, India. Tween^®^ 80 (97.3%), and Tween^®^ 20 (97.3%) were received as gift samples from Finar Chemical, Ahmedabad, India. All other reagents/chemicals used in this study are of analytical grade.

### 4.2. Drug Analysis

Quantification of Dolutegravir was performed using an HPLC system (Jasco LC–4000, Easton, MD, USA) integrated with a UV-visible detector, at a wavelength of 260 nm and internal standard (quinoxaline), as described in our previous article [53]. Gradient elution was used to separate Dolutegravir using a Discovery C18 HPLC column (250 mm × 4.6 mm, i.d, 5 μm) with acetonitrile and a 50 mM solution of acetate buffer (pH 4.5) as mobile phase, while the solvent flow rate was 1 mL/min [54]. Acetonitrile: acetate buffer (40:60) was used for the gradient elution until 4 min, after which a ratio of 70:30 was used. Furthermore, the validation of analytical methods was also carried out as per the procedure described in our earlier study [53].

### 4.3. Saturation Solubility Study

The solubility of Dolutegravir in different solvents such as oils (dill oil, castor oil, corn oil, and Capryol^®^ 90), surfactants (Tween^®^ 80, Labrafil^®^ M, Tween^®^ 20, and Labrasol^®^), and cosurfactants (Transcutol^®^ P, PEG 400, propylene glycol, and butyl carbitol) was assessed. Briefly, a vortex mixer was used to mix a surplus quantity of drugs with 1 mL of chosen solvents in stopper vials. The saturated solution was centrifuged at 3000 rpm for 10–15 min after being equilibrated for an entire night at ambient temperature. Following separation, the supernatant was diluted with methanol. The concentration of Dolutegravir in various solvents was assessed by HPLC.

### 4.4. Pseudo-Ternary Phase Diagram

The phase diagram was created using selected solvent constituents of oil (dill oil), surfactant (Tween^®^ 80), cosurfactant (Transcutol^®^ P), and water. A titration approach was used to obtain the concentration ranges that exist within the phase diagram of the nanoemulsion zone at room temperature [55]. Briefly, Tween^®^ 80 and Transcutol were mixed (S_mix_) in various ratios 1:2, 1:3, 2:1, and 3:1, respectively. The components were homogenized by gently heating each surfactant/cosurfactant mixture. To check the transparency, 0.1 mL from each mixture was diluted with 10 mL of methanol in a stopper conical flask and was kept aside for 2 h. The percentage transparency of the mixture was checked at 260 nm using methanol as a blank. The oil phase was then combined with aliquots of each S_mix_ in a stoppered glass vial at 25 °C. The oil to S_mix_ ratios used were in the range of 1:9 to 2:8, 3:7 to 4:6, 5:5, 6:4, 7:3, 8:2, and 9:1. Each combination of oil and S_mix_ was subjected to a separate slow titration with the aqueous phase. The percentage of each component present in the nanoemulsion was calculated before adding the aqueous phase. Water was added at an increment of 5% using a micropipette to produce a water content varying between 5% and 95%. For instance, the composition for preparing a pseudo-ternary diagram prepared using 1:9 ratios of oil and S_mix_ is given in Table S2. Visual observation was made following each injection of water to the oil: S_mix_ (Table S3). A separate ternary phase diagram was subsequently constructed using DPLOT software for each ratio of oil and S_mix_.

### 4.5. Preparation of Dolutegravir Nanoemulsion

Dolutegravir-loaded nanoemulsions were formulated by adding the required amount of water, dill oil, Tween^®^ 80, and Transcutol^®^ P. Dolutegravir (5% *w*/*v*) was dispersed in dill oil and added to the *S*_mix,_ with continuous mixing. The blend was titrated by adding drop-by-drop of water, and it was then stirred and vortexed to obtain the nanoemulsion spontaneously. The nanoemulsion (o/w) region of a formulation comprising water/S_mix_/oil system was assessed visually based on the physical state of the mixture at room temperature (25 ± 1 °C).

### 4.6. Optimization of Nanoemulsion

Finding the impact of formulation composition on particle size is the main goal of the statistical modeling of phase diagrams. The link between the size of nanoemulsion particles and the quantity of various constituents was determined using a simplex lattice design. An equilateral triangle in two dimensions represents the simplex lattice architecture for a three-component system (Figure S2). This method involved the plotting of pseudo-ternary phase diagrams and the evaluation of the particle size distribution at several locations inside the nanoemulsion zone. Seven formulations (F1–F7) were created, three at each of the three vertices (A, B, and C), three at the center point (AB, BC, and AC), and one at the halfway point between the vertices (ABC). The layout of the simplex lattice design is shown in Table 5.

### 4.7. Characterization of Nanoemulsions

#### 4.7.1. Percentage Transmittance and Phase Separation

Optical transparency of the nanoemulsions was evaluated based on percentage transmittance value. The percentage transmittance of all prepared nanoemulsions (F1–F7) was determined by a photometer (Photoelectric Colorimeter 113, Systronics, Ahmedabad, India) using a transparent cuvette. Each nanoemulsion system was centrifuged at 1107× *g* for 15 min and checked for phase separation [33].

#### 4.7.2. Particle Size Characterization

Using the Nano ZS90 (Malvern Instruments, Malvern, UK), the globule size, distribution, as well as polydispersity index of the prepared formulation were assessed. Briefly, several droplets of each test sample were poured into a cell while facing the laser light source. Backscatter detection was used to assess the rate of fluctuations in the intensity of scattered light, and the particle size was calculated [56]. After adequate dilution of nanoemulsions, the electrophoretic mobility values were assessed using the software DTS, version 4.1, to determine the zeta potential (Malvern, England, UK, 2009).

#### 4.7.3. Transmission Electron Microscopy (TEM)

Utilizing a TEM (Tecnai 20, Philips, Holland) with a 200 kV operating voltage and a 0.15 nm effective total resolution, the shape of nanoemulsions was characterized. The morphology and structure of the nanoemulsions were assessed after staining. The sample was then allowed to dry at ambient temperature.

#### 4.7.4. In Vitro Release

A vertical Franz diffusion cell with a 1.3 cm^2^ surface area (Orchid Scientific and Innovative India Ltd., Nashik, India) was used for the drug release experiments. In brief, nanoemulsion or gel (5 mg of Dolutegravir) was placed in a cellophane dialyzing membrane (MWCO 12–14 kDa). Artificial nasal fluid (pH 6.4) was used as the receiver media [57]. The receptor media were continuously agitated at 50 rpm while the temperature was maintained at 37 ± 0.5 °C [58]. At scheduled times (0.5, 1, 2, 4, 6, and 8 h), samples (1 mL) were taken out and replaced with the same amount of simulated nasal fluid. The amount of Dolutegravir released was determined using HPLC after being appropriately diluted.

#### 4.7.5. Statistical Analysis of the Data and Validation of the Model

Various formulations prepared based on simplex lattice design were statistically analyzed using design expert software (v.11.0). The multiple linear regression method was used to create polynomial models for all of the output responses that included interaction and quadratic terms. The software was used to create contour plots and three-dimensional (3D) response graphs. To assess the reliability of the model created, two random checkpoints were conducted throughout the whole spectrum of experimental domains. The response qualities that were experimentally obtained data were then quantitatively matched with those of the predicted values. Table 6 shows the details of checkpoint batches. The checkpoint formulation was coded as F8 and F9 and further evaluated for droplet size and release. From the results of the study, the checkpoint batches were validated using the polynomial equation.

### 4.8. Preparation of Dolutegravir Gel

Various gel formulations were developed by the cold method, which has been described before [59,60]. Table 7 shows the percentage of ingredients used for the in situ gels (B1-B6) preparation. Briefly, cold water was used to dissolve the specified amount of poloxamer 407 (20–22% *w*/*v*) and the blend was then kept at 4 °C to obtain a clear solution. Carbopol 934P, at the varying amount (0.1–0.5% *w*/*v*), was slowly added to the aforementioned poloxamer solution with continuous stirring. The obtained solution was added with optimized Dolutegravir (0.5% *w*/*v*)-loaded nanoemulsion (F3) and methylparaben as a preservative (0.05%). The preparation was constantly agitated with a magnetic stirrer to obtain a homogeneous solution. Control gel was prepared similarly by adding 0.5% *w*/*v* of the drug.

### 4.9. Evaluation of Formulated Gels

#### 4.9.1. Appearance

The preparation was assessed for transparency by visually checking under high illumination and observing against a black-and-white screen. Additionally, the formed gels were carefully examined for the existence of any particle matter and turbidity buildup [61].

#### 4.9.2. pH and Drug Content

Using a calibrated pH meter, the pH of different in situ gel formulations (B1–B6) was determined. To carry out the estimation of drug content, 1 g of gel was dispersed uniformly with mobile phase using a laboratory blender (EIE 405, EIE Instruments, Ahmedabad, India) for 10 min. The solution obtained was subsequently filtered using a 0.22 µm pore size filter membrane and the Dolutegravir content was quantified by HPLC.

#### 4.9.3. Measurement of Gelation Temperature

The visual observation approach, which is described elsewhere [42], was used to record the gelation temperature of formulations (B1–B6). Briefly, a specific amount of gel (5 mL) was placed in a glass vial and a magnetic bead was added. Samples were submerged in a thermostatically controlled water bath. The temperature was slowly raised. The temperature at which the bead stopped moving is considered gelation temperature.

#### 4.9.4. Viscosity

A Brookfield Viscometer was used to measure the viscosity of in situ gels (B1–B6). The temperature was set at 30 ± 1 °C, while the speed of the spindle was maintained at 20 rpm.

### 4.10. Ex Vivo Permeation

The drug diffusion potential of selected drug-loaded nanoemulsion in situ gels (B1) and Dolutegravir gel (control) was measured using the Franz diffusion cell apparatus mentioned in Section 4.7.4. Fresh nasal mucosa was isolated from slaughtered sheep’s nasal cavities, and it was then instantly frozen at −20 °C in a deep freezer [62]. The nasal membrane was kept in the space between two chambers of a diffusion cell. The mucosa layer was always in direct contact with the gel and artificial nasal fluid was in the receiving chamber [45]. The donor compartment was loaded with gel (B1, 1 g) containing 5 mg of Dolutegravir or a control. The receiver cell was stirred at 50 rpm, and the system’s temperature was set at 34 ± 0.5 °C. Samples collected at various intervals were injected into the HPLC system. Various permeation parameters were determined according to the literature [63,64].

### 4.11. Nasal Ciliotoxicity Studies

Freshly isolated sheep nasal mucosa was used to perform the ex vivo nasal ciliotoxicity study for the selected in situ formulation (BI) of Dolutegravir. Histopathological studies were conducted on three identically cut nasal mucosa samples (A, B, and C) and were placed separately on Franz diffusion cells to examine any potential toxicity on nasal tissues [65]. The entire study was performed at a temperature of 37 ± 1 °C. Sample A was treated with 0.5 mL of simulated nasal fluid. Sample B was treated with 0.5 mL of isopropyl alcohol to induce redness, and test sample C was treated with 0.5 mL of selected Dolutegravir-loaded nanoemulsion gel (B1). The mucosa was examined histologically using a hematoxylin-eosin staining solution after being exposed to nasal saline fluid for 6 h [66]. The microscopic image of stained slides was taken with 400 x magnification (ZEISS, Axioscope 5, Jena, Germany).

### 4.12. Animal Experiments

The Institutional Animal Ethical Review Board examined and authorized the methodology for animal testing (IAEC no. IP/PCEU/FAC/23/2018/03). Male Sprague Dawley rats weighing 250–300 g were used to estimate various pharmacokinetic properties. Six rats were used for each time point, and the animals were separated into two groups (Group I and Group II) at random. They were kept in individual cages in a temperature-controlled environment with a 12 h light/dark, and during the acclimation period, a standard feed and water were available at all times. Rats were given formulations after fasting for at least 12 h. Using the equation described in the scientific literature, the dose was determined from the 50 mg daily dose for humans [67]. Rats in Group I were given an intranasal dose of a chosen formulation (B1) containing 0.5% of *w*/*v* Dolutegravir (30 μL, 5 mg/kg or 150 µg/rat). Through the tail vein, group II animals received 0.15 mg/0.4 mL (5 mg/kg) of Dolutegravir in an intravenous solution made with 10% dimethyl sulfoxide, 40% PEG 300, 5% Tween^®^ 80, and 45% saline.

All rats were anesthetized with thiopental sodium (30 mg/kg) and samples (200 μL) were collected from the retro-orbital plexus at the predetermined time points. Precipitation of proteins in plasma was performed by adding a 50 mM acetate buffer (pH 4.5) [54]. Furthermore, brains from all the sacrificed animals were homogenized and subsequently extracted with acetonitrile at 4 °C for 5 min. Noncompartmental analysis was used to estimate the pharmacokinetic characters [68].

### 4.13. Stability Studies

The selected formulation (B1) of Dolutegravir-loaded nanoemulsion in situ gel formulation was assessed for its chemical and physical stability according to ICH guidelines [69]. Gels were placed at 25 ± 5 °C and 60% ± 5% RH for three months in a stability chamber. The stored gels were evaluated for various pharmaceutical properties.

## Figures and Tables

**Table 1:** Composition of design batches and responses of dependent variables.

Formulations	Dill Oil (%) X_1_	S_mix_ (%) X_2_	Water (%) X_3_	Particle Size (nm) Y_1_	% Drug Release in 8 h Y_2_
F1	12.5	17.5	70	240.53 ± 31.05	76.26 ± 5.25
F2	12.5	10	77.5	252.32 ± 29.45	70.05 ± 4.30
F3	5	25	70	106.36 ± 34.07	95.80 ± 4.11
F4	5	17.5	77.5	118.84 ± 31.24	86.00 ± 5.62
F5	7.5	20	72.5	206.60 ± 38.76	78.24 ± 4.82
F6	20	10	70	286.65 ± 41.68	61.08 ± 4.25
F7	5	10	85	184.86 ± 37.43	80.23 ± 5.18

**Table 2:** Validation of design model.

Formulation Code	Particle Size Y_1_		In Vitro Release Y_2_	
	Experimental Value	Predicted Value	Experimental Value	Predicted Value
F8	107.92 ± 26.48	109.30	94.4 ± 4.14	93.90
F9	120.66 ± 22.63	122.4	87.8 ± 3.97	88.3

**Table 3:** Physicochemical characteristics of in situ gels having Dolutegravir.

Formulation Code	pH	% Drug Content	Gelation Temperature (°C)	Viscosity (cP)
B1	5.81 ± 0.26	99.17 ± 1.65	30.26 ± 0.49	2965 ± 441
B2	5.52 ± 0.31	98.54 ± 2.76	29.62 ± 0.83	5102 ± 925
B3	5.25 ± 0.15	98.58 ± 1.27	28.81 ± 0.71	7006 ± 1238
B4	5.94 ± 0.26	98.49 ± 1.88	26.13 ± 0.61	3104 ± 536
B5	5.65 ± 0.44	98.43 ± 2.76	25.65 ± 0.96	5236 ± 811
B6	5.38 ± 0.34	98.99 ± 2.16	24.91 ± 0.38	7338 ± 1040

The value mentioned is average ± SD (n = 3).

**Table 4:** Measured pharmacokinetics properties following a single-dose of Dolutegravir (150 µg/rat) given by intravenous injection or selected gel (B1) by nasal route (n = 6).

Parameters	Brain		Plasma	
	In situ Nasal Gel-B1	IV Solution	In Situ Nasal Gel-B1	IV Solution
C_max_ (ng/mL)	2274.75 (±265.64) *	387.42 (±93.63)	261.99 (±112.61)	-
T_max_ (h)	1	2	2	-
AUC_0→t_ (ng·h/mL)	21,869.80 (±1814.35) *	4345.21 (±368.59)	4085.73 (±374.96) *	21,982.34 (±1689.98)
AUC brain/AUC plasma	5.35	0.20	-	-

* *p* < 0.0001, significantly different when compared with the intravenous (IV) solution.

**Table 5:** Design matrix of simplex lattice design with coded and uncoded values.

Formulations	Coded Value			Actual Value (%)		
	Dill Oil (X_1_)	S_mix_ [Tween^®^ 80: Transcutol (1:1)] (X_2_)	Water (X_3_)	Dill Oil (X_1_)	S_mix_ (X_2_)	Water (X_3_)
F1	0.496	0.504	0	12.5	17.5	70
F2	0.505	0	0.495	12.5	10	77.5
F3	0	1	0	5	25	70
F4	0	0.501	0.499	5	17.5	77.5
F5	0.314	0.386	0.3	7.5	20	72.5
F6	1	0	0	20	10	70
F7	0	0	1	5	10	85

**Table 6:** Composition of checkpoint formulation.

Formulation	Coded Value			Actual Value (%)		
	Dill Oil (X_1_)	S_mix_ (X_2_)	Water (X_3_)	Dill Oil (X_1_)	S_mix_ (X_2_)	Water (X_3_)
F8	0.026	0.940	0.034	5.2	23.5	71.3
F9	0.05	0.69	0.26	10	17.25	72.75

**Table 7:** Compositions of nasal in situ gels of Dolutegravir.

Composition	B1	B2	B3	B4	B5	B6
Nanoemulsion-loaded Dolutegravir (%)	0.5	0.5	0.5	0.5	0.5	0.5
Poloxamer 407 (%)	20	20	20	22	22	22
Carbopol 934P (%)	0.1	0.3	0.5	0.1	0.3	0.5
Methyl paraben (%)	0.05	0.05	0.05	0.05	0.05	0.05
Distilled water (%)	Up to 100	Up to 100	Up to 100	Up to 100	Up to 100	Up to 100

## Data Availability

The data presented in this study are contained within the article.

## Supplementary Materials

The following supporting information can be downloaded at: https://www.mdpi.com/article/10.3390/gels9020130/s1. Figure S1: percentage of Dolutegravir released from nanoemulsions (F1–F7). The value mentioned are average ± SD (n = 6); Figure S2: simplex lattice design. Table S1: percentage transparency at various ratios of surfactant and cosurfactant; Table S2: composition of the pseudo-ternary phase diagram (oil and S_mix_ ratio of 1:9); and Table S3: visual observation during aqueous phase titration for phase diagram construction.
