# Supplementary material for: Intranasal Administration of Dolutegravir-Loaded Nanoemulsion-Based In Situ Gel for Enhanced Bioavailability and Direct Brain Targeting
Source: Gels. 2023 Feb 3;9(2):130. doi: 10.3390/gels9020130 (PMC9956165; doi:10.3390/gels9020130)
Supplement: Supplementary file 1 [file gels-09-00130-s001.zip › gels-2201249-supplementary.pdf]

gels

MDPI

Article

Intranasal Administration of Dolutegravir-Loaded Nanoemulsion-Based In Situ Gel for Enhanced Bioavailability and Direct Brain Targeting

Anroop B. Nair [1,*,†], Sunita Chaudhary [2,†], Shery Jacob [3], Dhwani Patel [2], Pottathil Shinu [4], Hiral Shah [2], Ankit Chaudhary [5], Bandar Aldhubiab [1], Rashed M. Almuqbil [1], Ahmed S. Alnaim [1], Fatemah Alqattan [1] and Jigar Shah [6,*]

Table S3. Percentage transparency at various ratios of surfactant and cosurfactant.

Surfactant : Cosurfactant	% Transparency
1:1	99.3%
1:2	95.9%
1:3	79.1%
2:1	64.5%
3:1	69.2%

Table S2. Composition of pseudo ternary phase diagram (oil and Smix ratio of 1:9).

Oil (µL)	Smix (µL)	Water (µL)	Total (µL)	Oil %	Smix %	Water %
10	90	10	110	9.09	81.82	9.09
10	90	20	120	8.33	75.00	16.67
10	90	25	125	8.00	72.00	20.00
10	90	35	135	7.41	66.67	25.93
10	90	45	145	6.90	62.07	31.03
10	90	55	155	6.45	58.06	35.48
10	90	65	165	6.06	54.55	39.39
10	90	80	180	5.56	50.00	44.44
10	90	100	200	5.00	45.00	50.00
10	90	120	220	4.55	40.91	54.55
10	90	150	250	4.00	36.00	60.00
10	90	185	285	3.51	31.58	64.91
10	90	235	335	2.99	26.87	70.15
10	90	300	400	2.50	22.50	75.00
10	90	400	500	2.00	18.00	80.00
10	90	550	650	1.54	13.85	84.62
10	90	900	1000	1.00	9.00	90.00
10	90	2000	2100	0.48	4.29	95.24

Table S3. Visual observation during aqueous phase titration for phase diagram construction.

Volume of aqueous phase	Oil: S_{mix} ratio								
	**1:9**	**2:8**	**3:7**	**4:6**	**5:5**	**6:4**	**7:3**	**8:2**	**9:1**
10 µL									
10 µL									
5 µL	NE								
10 µL	NE								
10 µL	NE		NE	NE				NE	
10 µL	NE	NE	NE	NE	NE		NE	NE	
10 µL	NE	NE	NE	NE	NE	NE	NE	NE	NE
15 µL	NE	NE	NE	NE	NE	NE	NE	NE	NE
20 µL	NE	NE	NE	NE	NE	NE	NE	NE	NE
20 µL	NE	NE	NE	NE	NE	NE	NE	NE	NE
30 µL	NE	NE	NE	NE	NE	NE	NE	NE	NE
35 µL	NE	NE	NE	NE	NE	NE	NE	NE	NE
50 µL	NE	NE	NE	NE	NE	NE	NE	NE	NE
65 µL	NE	NE	NE	NE	NE	NE	NE	NE	NE
100 µL	NE	NE	NE	NE	NE	NE	NE	NE	NE
150 µL	NE	NE	NE	NE	NE	NE	NE	NE	NE
350 µL	NE	NE	NE	NE	NE	NE	NE	NE	NE
1100 µL	NE	NE	NE	NE	NE	NE	NE	NE	NE

NE, nanoemulsion

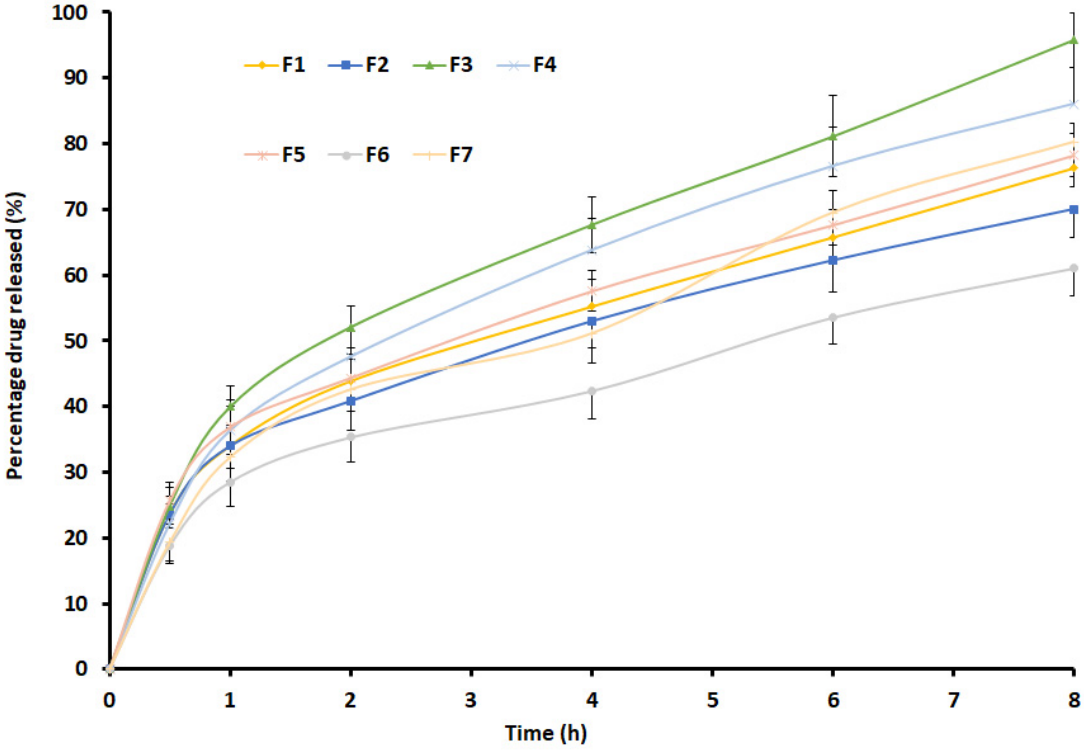

Figure S1. Percentage Dolutegravir released from nanoemulsions (F1-F7). The value mentioned are average ± SD (n = 6).

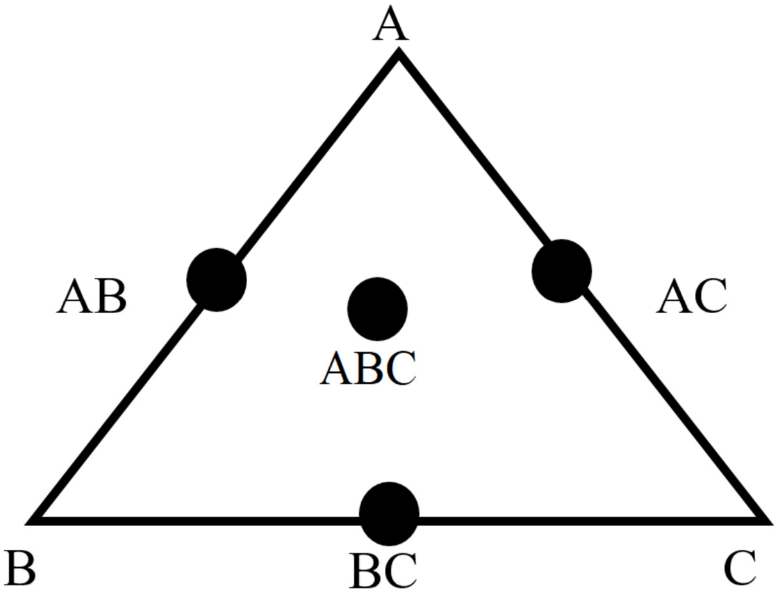

Figure S2. Simplex lattice design.
